# Local dominance of exotic plants declines with residence time: a role for plant–soil feedback?

**DOI:** 10.1093/aobpla/plv021

**Published:** 2015-03-13

**Authors:** Tanja A.A. Speek, Joop H.J. Schaminée, Jeltje M. Stam, Lambertus A.P. Lotz, Wim A. Ozinga, Wim H. van der Putten

**Affiliations:** 1 Plant Research International, Wageningen University and Research Centre, Droevendaalsesteeg 1, 6708 PB Wageningen, The Netherlands; 2 Laboratory of Nematology, Wageningen University and Research Centre, Droevendaalsesteeg 1, 6708 PB Wageningen, The Netherlands; 3 Department of Terrestrial Ecology, Netherlands Institute of Ecology (NIOO-KNAW), Droevendaalsesteeg 10, 6708 PB Wageningen, The Netherlands; 4 Centre for Ecosystem Studies, Wageningen University and Research Centre, Droevendaalsesteeg 3a, 6708 PB Wageningen, The Netherlands; 5 Department of Ecology, Aquatic Ecology and Environmental Biology Research Group, Radboud University Nijmegen, Heyendaalseweg 135, 6525 AJ Nijmegen, The Netherlands; 6 Laboratory of Entomology, Wageningen University and Research Centre, Droevendaalsesteeg 1, 6708 PB Wageningen, The Netherlands

**Keywords:** Exotic species, introduced species, local dominance, macro ecology, residence time, soil-borne enemy

## Abstract

Recent studies have shown that introduced exotic plant species may be released from their native soil-borne pathogens, but that they become exposed to increased soil pathogen activity in the new range when time since introduction increases. Other studies have shown that introduced exotic plant species become less dominant when time since introduction increases, and that plant abundance may be controlled by soil-borne pathogens; however, no study yet has tested whether these soil effects might explain the decline in dominance of exotic plant species following their initial invasiveness. Here we determine plant–soil feedback of 20 plant species that have been introduced into The Netherlands. We tested the hypotheses that (i) exotic plant species with a longer residence time have a more negative soil feedback and (ii) greater local dominance of the introduced exotic plant species correlates with less negative, or more positive, plant–soil feedback. Although the local dominance of exotic plant species decreased with time since introduction, there was no relationship of local dominance with plant–soil feedback. Plant–soil feedback also did not become more negative with increasing time since introduction. We discuss why our results may deviate from some earlier published studies and why plant–soil feedback may not in all cases, or not in all comparisons, explain patterns of local dominance of introduced exotic plant species.

## Abstract (precis)

Exotic plant species have shown boom-bust patterns, first becoming invasive, but then over a longer time period declining in dominance. Exotic plant species may escape from their native enemies, but might become increasingly exposed to enemies in the new range as time since introduction increases. We investigated whether soil interactions could explain a pattern in the Netherlands where exotic plant species with a longer residence time are less dominant, by performing a plant soil feedback experiment. We found no evidence that plant-soil interactions explain this pattern.

## Introduction

An important challenge for invasion ecologists is to predict the course of invasions of introduced exotic species. This requires insight in the factors that may control the abundance and dominance of species in both their native and new ranges. It has been well established that regional distribution of exotic plant species increases with residence time (Pyšek *et al.* 2004; Hamilton *et al.* 2005; Wilson *et al.* 2007; Milbau and Stout 2008; Bucharova and van Kleunen 2009; Gassó *et al.* 2009). It has also been argued that increased residence time may result in lower local dominance and invasiveness (Carpenter and Cappuccino 2005; Hawkes 2007; Speek *et al.* 2011). Local dominance of introduced exotic plant species may be, at least in part, driven by interactions with soil biota, including effects of soil-borne enemies and symbionts (Inderjit and van der Putten 2010). The question that we address in the present study is how residence time and local dominance of exotic plant species may relate to enemy impact of the soil biota. Ultimately, this information may be used to enhance predictions on the course of invasiveness of introduced exotic plant species.

A possible explanation for lower local dominance of introduced exotic plant species with a long residence time is that enemy species may increasingly adapt and accumulate when time of exposure to the new hosts increases (Hawkes 2007; Diez *et al.* 2010; Dostál *et al*. 2013). Both aboveground (Bentley and Whittaker 1979; Gange and Brown 1989) and belowground (van der Putten *et al*. 1993; Klironomos 2002; Mangan *et al*. 2010; Johnson *et al*. 2012) enemies may control local plant dominance. Release from natural enemies by introduction to a new range has been proposed to enhance the performance of species and, therefore, their invasiveness (Elton 1958; Keane and Crawley 2002). This ‘enemy release hypothesis’ (Keane and Crawley 2002) has been supported by surveys showing that introduced plant species have fewer enemies in their novel than native range (e.g. Mitchell and Power 2003).

Thus far, the majority of research on enemy release of exotic plant species has been dedicated to aboveground enemies. However, an increasing amount of studies is showing that introduced exotic plant species can be released from native soil-borne enemies as well (Reinhart *et al*. 2003, 2010; Callaway *et al*. 2004; Gundale *et al*. 2014). Introduced exotic plant species suffer less from soil-enemies of the invaded range than congeners that are native in that range (Maron and Vilà 2001; Agrawal *et al*. 2005; Engelkes *et al*. 2008).

The change in performance of exotic species with progressing residence time has been described for several invaders (Simberloff and Gibbons 2004). Loss of exotic dominance might be caused by evolutionary adaptation of enemies in the new range to the introduced plant species (Müller-Schärer *et al*. 2004). Such adaptive potential may be deduced from reported higher frequencies of specialist compared with generalist herbivores (Andow and Imura 1994), higher exposure (Mitchell *et al*. 2010) and higher impact (Hawkes 2007) of enemies on crop and exotic plant species in relation to increasing residence time. Similarly, in New Zealand plant–soil feedback of 12 exotic plant species related negatively to their residence time (Diez *et al*. 2010) and in the Czech Republic giant hogweed (*Heracleum mantegazzianum*) developed negative feedback effects from the soil biota in fields that had been colonized for some decades (Dostál *et al*. 2013). However, in these latter studies, increased enemy exposure has not yet been related to local dominance of the exotic plant species, which is the key aim of the present study.

A recent analysis established that exotic plant species with a long residence time in The Netherlands have lower local dominance than recently introduced species (Speek *et al*. 2011). Until now, no study has related such patterns in local dominance to plant–soil feedback effects. Therefore, in the present study, we determine how residence time, local dominance and soil pathogen effects to exotic species may relate to each other. We tested soil pathogen effects by plant–soil feedback approach (Bever *et al*. 1997), which is a way to experimentally integrate all positive and negative interactions between plants and the soil biota. We first tested the hypothesis that species with a longer residence time have a more negative plant–soil feedback (Diez *et al*. 2010). Then, we tested the hypothesis that species with a more positive plant–soil feedback have a higher local dominance (Klironomos 2002).

## Methods

### Data on plants, their residence time and local dominance

Data on residence time were derived from information on the period of naturalization according to the standard list of the Dutch flora (Tamis *et al*. 2004). Data on local dominance were derived from the Dutch Vegetation Database (Schaminée *et al*. 2007), containing over 500 000 vegetation records including data on local species cover in plots varying from 1 by 1 m^2^ to 10 by 10 m^2^. Plot sizes used for recording depended on the characteristics of vegetation, for example largest plot sizes were used for forests. Data on plant species cover were used to calculate local dominance as [the number of vegetation records with that species having >10 per cent ground cover/the total number of vegetation records with that plant species] × 100 % (Speek *et al*. 2011). Therefore, local dominance expresses the frequency of how often a plant species has a minimum cover of 10 %, when present in the vegetation record. In order to exclude recorder bias, for example due to avoiding taking records of vegetation heavily invaded by exotic plant species, we used expert judgment to check and where necessary adjust the cover data (Speek *et al*. 2011).

### Soil feedback experiment

We used a selection of 20 introduced exotic plant species in The Netherlands for a plant–soil feedback experiment (Supplements). The selection of 20 plant species was based on a number of criteria. First, we excluded woody species, because the length of the plant–soil feedback is too limited for capturing a substantial part of the life cycle of trees. We then selected as many as possible plant species from riverine areas in order to be able to use the same soil origin for all plant species. Finally, the selection was limited as the seeds of some plant species did not germinate. Seeds had been collected by specialized seed companies that collect seeds locally, or by ourselves or colleagues.

Of the 20 plant species, 14 occur in the Millingerwaard (Dirkse *et al*. 2007), a riverine floodplain area of 700 hectares. Millingerwaard is a nature reserve in the riverine floodplain of the river Waal, which is in the southern branch of the Rhine river in The Netherlands (51°87′N, 6°01′E). Three other species occur near or in other riverine areas in The Netherlands and the remaining three occur outside riverine areas. We collected soil from the Millingerwaard area, instead of from a larger variety of sites, as soil from a variety of sites would have introduced additional variation due to soil type, fertility, pH etc. All plant species were forbs that varied in local dominance from 5 to 38 % and in residence time from 75 to 400 years.

Seeds were germinated on glass beads placed in demineralized water. Germination was carried out in transparent plastic containers of 17 × 12 × 5 cm that were placed under conditions of 16 h 22 °C in the light (day) and 8 h 10 °C in the dark (night). *Xanthium strumarium* seeds were germinated at a higher temperature: 16 h 32 °C and 8 h 20 °C. Germinated seedlings were stored at 4 °C and 10/14 h light/dark until transplantation in soil, to ensure equal sizes at start of the experiment. Soil was collected from five random locations in Millingerwaard. Soil to be used as inoculum was collected in October 2010, prior to the first phase of the experiment. Soil from the five sampling locations was sieved (mesh size 5 mm) to remove coarse roots, stones and other large particles, and subsequently homogenized. The bulk soil was collected in January 2010, sterilized by gamma irradiation (25 kGray) and stored in sealed plastic bags at 4 °C until use.

The sensitivity of exotic plant species to soil-borne enemies was determined in a so-called two-phase plant–soil feedback experiment (Bever *et al*. 1997). In the first phase, which started from one pooled sample, the seedlings were grown to condition the field soil. In that phase, soil biota that can grow on resources provided by that particular plant species are enumerated (Grayston *et al*. 1998; Kowalchuk *et al*. 2002). In the second phase, we kept all replicates of own soil separate. In order to do so, the soil of each pot was split into two halves: one half was used as own soil, whereas the other half was mixed with halves of all other replications and species, to be used as away soils. The replicates of the mixed soil were not kept intact, because there was no relationship between replicate 1 conditioned by species A or B. Comparing plant performances in own and mixed soils enabled us to make a home (own) versus away (mixed) comparison, which is a less sensitive and ecologically more realistic method of detecting plant–soil feedback effects than a comparison of non-sterilized versus sterilized soil (Kulmatiski *et al*. 2008). In the final analysis, plant species was the unit of replication.

For the first—conditioning—phase, bulk soil and inoculum were mixed at a ratio of 4 : 1, with a total of 1200 g soil per pot on a dry weight basis. Pots of 1.3 L were used. For the second—feedback—phase, ‘own soil’ and ‘mixed soil’ were homogenized with sterilized bulk soil at a ratio of 1 : 1 in order to keep pot volumes equal between the two feedback phases. For each plant species, we had five independent replicates with own and five with mixed soil. Every pot contained three seedlings, except *Amaranthus retroflexus* that was planted as two seedlings per pot due to poor germination of the seeds. Dead seedlings were replaced until the first week after transplanting. Greenhouse conditions were maintained at 60 % RH, day temperature 21 °C, night temperature 16 °C. Daylight was supplemented with lamps (SON-T Agro, 225 µmol^−1^ m^−2^), to ensure a minimum of 16 h light per day.

Before planting, the water content in each pot was set at 20 % (w/w). Plants were supplied with water three times a week and once a week the water content was re-set to 20 % by weighing. Plants received 10 mL of 0.5 strength Hoagland per pot in weeks 2, 3 and 4, and 20 mL in weeks 5 and 6 after transplanting in order to meet the increasing demand. Plants were harvested 6 weeks after planting. The length of growth was the same for both phases, which is relatively short, but ample for testing feedback responses (van der Putten *et al*. 1988). When harvesting, shoots of the three (or two) plants per pot were clipped at ground level, pooled, dried in paper bags at 75 °C until constant weight and weighed, so that biomass data per pot were obtained.

### Statistical analysis

The effect of soil feedback on shoot and root biomass was calculated as ln[(biomass in own soil)/(biomass in mixed soil)] (Brinkman *et al*. 2010). We assigned pairs of own soil with mixed soil randomly. To analyse whether residence time or local dominance could explain mean shoot and root feedback responses, we used linear models. The unit of replication was the plant species. For residence time we used models with a normal distribution, for local dominance we used models with a binomial distribution and a logit link, with binomial totals set to 50 % (the highest value in our dataset).

We analysed which traits and other factors related best to residence time by a model selection procedure within a linear model with a normal distribution. Thus, we selected the best minimal adequate model with the lowest Akaike Information Criterion value from all possible subsets. Although time and dominance were related, the relation of a trait or other factor to residence time may not necessarily imply that there is a relation with local dominance as well. Therefore, the factors in the best minimal adequate model were added to a generalized linear model with residence time explaining local dominance. By adding each factor separately, we analysed which one significantly changed the model. Factors that affected the model were likely to be a better explanation for variation in local dominance than residence time. For explaining local dominance we used a binomial distribution with a logit link, binomial totals set at 50 and accounting for overdispersion. All analyses were done in Genstat, version 14.

## Results

**Figure 1.**
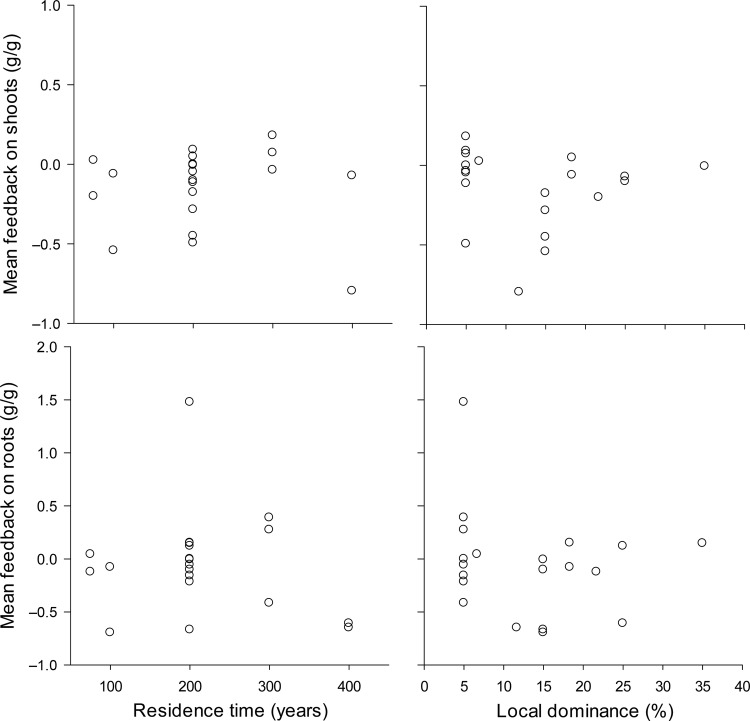
Mean soil feedback effect on the biomass of shoots and roots in relation to the residence time or the local dominance of naturalized exotic plant species in The Netherlands. Each circle represents a different plant species.

Opposite to our hypothesis, we found neither a significant relationship between residence time and plant–soil feedback of the exotic plant species, nor for shoots (*F* = 0.10, *t*_18_ = −0.32, *P* = 0.751, Fig. 1) and for roots (*F* = 0.41, *t*_18_ = −0.64, *P* = 0.529). Local plant dominance also did not relate to the feedback effect on shoots (*F* = 0.09, *t*_18_ = −0.31, *P* = 0.763) or roots (*F* = 0.73, *t*_18_ = −0.85, *P* = 0.404). Excluding species from riverine habitats, which may not be responsive to soil biota from that habitat, or Fabaceae species, which may have a different feedback due to symbiosis with rhizobia did not alter the significance of the results (data not shown). Therefore, our hypotheses that species with a longer residence time have a more negative plant–soil feedback, and that species with a less negative or more positive plant–soil feedback have a higher local dominance were not supported.

## Discussion

In our study we tested the hypotheses that species with a longer residence time have a more negative plant–soil feedback and that species with a less negative, or more positive plant–soil feedback have a higher local dominance. We used an experimental approach to measure soil-borne enemy impact by plant–soil feedback approach. However, opposite to a study from New Zealand (Diez *et al*. 2010), and to a study on introduced *H. mantegazzianum* in the Czech Republic (Dostál *et al*. 2013) we did not find such a relationship between time since introduction of 20 exotic plant species in the Dutch flora and plant–soil feedback.

There are several possible explanations for these results. Our results could indicate that not all introduced exotic plant species develop negative plant–soil feedback when time since introduction increases. In the field, other ecological processes may be influencing community composition and aboveground interactions can either increase or decrease with the strength of the belowground interactions. Another possible explanation concerns the choice of soils for the plant–soil feedback experiment. We have chosen soils from areas where most exotic plant species may occur, but we did not use soils from the root zone of particular populations. This approach has led to marked differences in plant–soil feedback between natives and exotics (van Grunsven *et al*. 2007; Engelkes *et al*. 2008); however, it has resulted in scattered results when testing soil responses across an entire native range (van Grunsven *et al*. 2010).

The results may also depend on the relatively short conditioning and testing phases of 6 weeks each. Test phases of 6 weeks can detect feedback effects (van der Putten *et al*. 1988). Longer test periods may even result in pot limitations, which may obfuscate results. Conditioning for 6 weeks will have been relatively short, but to our experience this is possible when adding soil inocula to sterilized soil, as has been done in the present study.

Our use of pooled soils as ‘away’ treatment may have provided a conservative estimate of plant–soil feedback effects, because of reducing variances. Nevertheless, since we did not find significant relationships with time since abandonment, or local dominance, our results show that even with a highly sensitive test still no relationship could be detected between time since introduction, or local dominance and plant–soil feedback. Mixing soils from all plant species to produce ‘away’ soils could theoretically have led to single pathogens dominating the entire away soil community. However, a previous addition study using a variety of amounts of soil inocula showed that soil feedback effects increased gradually with the amount of inoculum added (van der Putten *et al*. 1988), which does not point at a disproportional role of pathogens from single plant species in the away soil mixtures.

Plant–soil biota interactions are highly local (Levine *et al.* 2006; Bezemer *et al*. 2010; Genung *et al*. 2012), and adaptation of soil organisms to new plant species does not take place at a national, but at a local scale through direct interactions between plant roots and the soil biota (Schweitzer *et al*. 2008; Lankau *et al*. 2009; Lau and Lennon 2011, 2012). As the feedback was estimated at a regional scale, also the local dominance was measured at a regional scale (first occurrence in The Netherlands). Using first occurrence in a larger region as an estimate of residence time could result in an over-estimation of the local residence time. On the other hand, the study from New Zealand (Diez *et al*. 2010) also used data on residence time for the entire country and not specifically for the sites from which the soil has been collected.

We expected plant–soil feedback to be negatively related to local dominance (Klironomos 2002; Mangan *et al*. 2010). However, in our study we did not observe such an inverse relationship. A possible explanation is that previous studies by Klironomos (2002) and Mangan *et al*. (2010) on dominance-feedback relationships have been based on native species, and that these relations may differ when considering exotic species. Moreover, we used dominance estimates averaged across the entire Netherlands (Speek *et al*. 2011), which differs from the local dominance estimates as used in other studies (e.g. Klironomos 2002). National estimates (in the case of The Netherlands concerning an area of appr. 150 × 300 km) will not provide accurate information about the local dominance of exotics in the riverine ecosystem where the soil for testing plant–soil feedback originated from. Therefore, it is possible that soil origin and plant dominance data were not well linked to each other, or that a relationship between plant–soil feedback and dominance works out differently for exotic plant species than for natives. Alternatively, our study may add to other examples where plant dominance does not relate to plant–soil feedback (Reinhart 2012).

An alternative explanation for the rejection of our hypotheses could be that the evolutionary dynamics leading to increased enemy pressure on exotic plant species is not strong enough to result in a change in mean local dominance. Meta-analyses have shown that a general pattern of decreased enemy numbers on exotic species in the novel range was not reflected in a general pattern of higher plant performance (Chun *et al*. 2010). Adaptation can occur both at the soil species level but also at the plant species level. This adaptation at two fronts is likely to result in a mixed general outcome. Moreover, while local dominance has been assumed to increase after introduction to a new range (Keane and Crawley 2002), recent work has shown that most species have the same dominance in both their introduced and native ranges (Firn *et al*. 2011). Clearly, local dominance is a complex trait, with a high variation both between and within species that can be influenced by a large number of ecological processes.

## Conclusions

We found no support for the hypothesis that the negative relationship between residence time and local dominance of exotic species in The Netherlands is caused by an increase in negative plant–soil feedback. It may be that data on residence time, dominance, enemy exposure and impact need to be collected all from the same area, or that different choices in plant–soil feedback approach need to be made (e.g. longer conditioning and/or feedback phases, a more sensitive ‘away’ soil treatment). Alternatively, it might be better to track single species across an introduction gradient (Lankau *et al*. 2009; Lankau 2011). It could also mean that not all introduced exotic plant species develop negative plant–soil feedback when time since introduction increases or that the hypothesized effect of increasing enemy pressure on dominance of introduced exotic plant species might not be strong enough to emerge from examining a large diversity of species across a variation of locations. Therefore even though we are aware of weaknesses of our paper (aspects of the experimental design that were not ideal, for example sampling of soil from one location that did not include all of the study species, pooling ‘away’ soils, method of pairing of home and away pots to calculate response ratios), our results may add to the debate on change in invasiveness of exotic plant species after introduction.

## Sources of Funding

T.A.A.S. and L.A.P.L. were funded by the former Dutch Ministry of Agriculture, Nature and Food Quality, FES-programme ‘Versterking Infrastructuur Plantgezondheid’. W.H.v.d.P. was supported by ALW-Vici grant (number 389 865.05.002). W.A.O. was supported by the Dutch Science Foundation (NWO Biodiversity Works).

## Contributions by the Authors

All authors contributed to the experimental set up and commented on the manuscript, T.A.A.S., J.M.S. and W.H.v.d.P. performed the experiment, T.A.A.S. performed all analyses, J.H.J.S. and W.A.O. provided data on local dominance of plant species, T.A.A.S. and W.H.v.d.P. wrote the paper.

## Conflict of Interest Statement

None declared.

## Supporting Information

The following additional information is available in the online version of this article –

**Table S1.** Plant species naturalized in The Netherlands that were used in soil–plant feedback experiments. Occurrence in Millingerwaard (area where soil was collected) is based on maps in Dirkse *et al.* 2007. + does occur in Millingerwaard; 0 does not occur in Millingerwaard but does occur in other floodplains in The Netherlands; − does not occur in Millingerwaard or other floodplains in The Netherlands.

Additional Information
