# Supplementary material for: Local dominance of exotic plants declines with residence time: a role for plant–soil feedback?
Source: AoB Plants. 2015 Mar 13;7:plv021. doi: 10.1093/aobpla/plv021 (PMC4408614; doi:10.1093/aobpla/plv021)
Supplement: Additional Information [file supp_7_plv021_index.html]

Local dominance of exotic plants declines with residence time: a role for plant–soil feedback? — Additional Information 

# Local dominance of exotic plants declines with residence time: a role for plant–soil feedback?

## Additional Information

Additional Information

**Files in this Data Supplement:**

- Supplementary Table 1 - Doc file
