# Supplementary material for: Local dominance of exotic plants declines with residence time: a role for plant–soil feedback?
Source: AoB Plants. 2015 Mar 13;7:plv021. doi: 10.1093/aobpla/plv021 (PMC4408614; doi:10.1093/aobpla/plv021)
Supplement: Additional Information [file supp_plv021_plv021supp.docx]

Supplements

Plant species naturalized in the Netherlands that were used in soil-plant feedback experiments.

Occurrence in Millingerwaard (area where soil was collected) is based on maps in Dirkse et al. 2007. + does occur in Millingerwaard; 0 does not occur in Millingerwaard but does occur in other floodplains in the Netherlands; - does not occur in Millingerwaard or other floodplains in the Netherlands.

| **Plant species** | **Family** | **Local dominance (%)** | **Residence time (year)** | **Occurs in Millinger-waard** |
| --- | --- | --- | --- | --- |
| *Allium carinatum* | Liliaceae | 5.00 | 200 | - |
| *Amaranthus blitoides* | Amaranthaceae | 15.00 | 100 | + |
| *Angelica archangelica* | Apiaceae | 18.33 | 100 | + |
| *Anthemis tinctoria* | Asteraceae | 11.67 | 400 | 0 |
| *Berteroa incana* | Brassicaceae | 25.00 | 200 | + |
| *Cymbalaria muralis* | Scrophulariaceae | 25.00 | 400 | + |
| *Datura stramonium* | Solanaceae | 5.00 | 300 | + |
| *Geranium pyrenaicum* | Geraniaceae | 5.00 | 200 | + |
| *Hieracium amplexicaule* | Asteraceae | 35.00 | 200 | 0 |
| *Medicago sativa* | Fabaceae | 15.00 | 200 | + |
| *Potentilla recta* | Rosaceae | 5.00 | 200 | + |
| *Salvia verticillata* | Lamiaceae | 15.00 | 200 | 0 |
| *Scrophularia vernalis* | Scrophulariaceae | 5.00 | 300 | 0 |
| *Senecio inaequidens* | Asteraceae | 21.67 | 75 | + |
| *Sisymbrium altissimum* | Brassicaceae | 15.00 | 200 | + |
| *Sisymbrium orientale* | Brassicaceae | 5.00 | 200 | + |
| *Solidago canadensis* | Asteraceae | 18.33 | 200 | + |
| *Tragopogon porrifolius* | Asteraceae | 5.00 | 300 | + |
| *Vicia villosa* | Fabaceae | 5.00 | 200 | + |
| *Xanthium strumarium* | Asteraceae | 6.67 | 75 | - |
